# Structural Characterization of Polysaccharides from *Coriandrum sativum* Seeds: Hepatoprotective Effect against Cadmium Toxicity In Vivo

**DOI:** 10.3390/antiox12020455

**Published:** 2023-02-10

**Authors:** Manel Sfar, Ghada Souid, Fahad M. Alminderej, Zeineb Mzoughi, Yassine El-Ghoul, Christophe Rihouey, Didier Le Cerf, Hatem Majdoub

**Affiliations:** 1 Laboratory of Interfaces and Advanced Materials, Faculty of Sciences of Monastir, University of Monastir, Monastir 5000, Tunisia; 2 Research Unit: Viral Genomic and Antiviral Strategy (UR 17ES 30), University of Monastir, Higher Institute of Biotechnology of Monastir, Monastir 5000, Tunisia; 3 Department of Chemistry, College of Science, Qassim University, Buraidah 51452, Saudi Arabia; 4 Textile Engineering Laboratory, University of Monastir, Monastir 5019, Tunisia; 5 Normandie University, UNIROUEN, INSA Rouen, CNRS, PBS, UMR 6270 & FR 3038, 76000 Rouen, France

**Keywords:** *Coriandrum sativum*, polysaccharide, physicochemical characterizations, antioxidants, hepatocytotoxicity

## Abstract

*Coriandrum sativum* is one of the most widespread curative plants in the world, being vastly cultivated in arid and semi-arid regions as one of the oldest spice plants. The present study explored the extraction of polysaccharides from Coriandrum sativum seeds and the evaluation of their antioxidant potential and hepatoprotective effects in vivo. The polysaccharide from coriander seeds was extracted, and the structural characterization was performed by FT-IR, UV–vis, DSC, NMR (1D and 2D), GC-MS, and SEC analysis. The polysaccharide extracted from *Coriandrum sativum* (CPS) seeds was characterized to evaluate its antioxidant and hepatoprotective capacities in rats. Results showed that CPS was composed of arabinose, rhamnose, xylose, mannose, fructose, galactose, and glucose in molar percentages of 6.2%, 3.6%, 8.8%, 17.7%, 5.2%, 32.9%, and 25.6%, respectively. Further, CPS significantly hindered cadmium-induced oxidation damage and exercised a protective effect against Cd hepatocytotoxicity, with a considerable reduction in MDA production and interesting CAT and SOD enzyme levels. Results suggest that CPS might be employed as a natural antioxidant source.

## 1. Introduction

Cadmium (Cd) is deemed one of the most predominant toxic metals in hydrous and terrestrial environments, owing to its toxicity, non-degradable property, and long half-life. Cd has been categorized as a nephrotoxic, neurotoxic/carcinogenic, and hepatotoxic heavy metal [1,2]. The xenobiotic was also described as a potent inducer of cytotoxicity and genotoxicity on cultured cells. Cadmium toxicity has the potential to stimulate lipid peroxidation and the generation of reactive oxygen species [3].

The liver is a basic organ that metabolizes exogenous and endogenous substances. Liver harm, a prevalent health problem, might result from drugs, chemicals, alcohol, exogenous substances, and infections generating the impairment of these crucial metabolic functions. Because chemical therapies, such as vaccines, antiviral medications, and steroids, have potentially adverse side effects, plant and herbal products with hepatoprotective and antioxidant properties with greater therapeutic efficacy and less toxicity are needed as substitutes [4]. Cadmium-induced hepatotoxicity is the most common model system used to estimate plant extracts/drugs’ hepatoprotective effects. Varied studies revelated that Cd was qualified as a liver lesion inducer with an interesting reducer of catalase (CAT) and reduced glutathione (GSH) content and histopathological alternations [5].

Curative, healing, and medicinal plants have been discovered and employed in traditional medicine practices since prehistoric times. They are often used owing to their low cost and milder side effects. Additionally, they are an effective source of natural antioxidant compounds, including polyphenols and polysaccharides [6]. Many recent research studies have reported the great development that natural polysaccharides have witnessed due to their various biological effects, including antioxidant, antimicrobial, antidyslipidemic, antidiabetic, and anticancer activities [7,8,9,10]. Coriander (*Coriandrum sativum* L.) is a medicinal and culinary plant of the Apiaceae family. It is commonly utilized as a relish or spice in the Mediterranean region and known as a source of essential oil and fatty acids. This plant has economic importance as it has been widely applied as a flavoring agent, in cosmetic products, and in perfume compositions. Coriander seed is a popular spice widely documented as a therapeutic agent in traditional medicine, mainly for the treatment of diabetes [11], heart and kidney problems, and gastrointestinal disorders [12], as well as to fight against worms, joint pain, and rheumatism [13]. *Coriandrum sativum* consists of several constituents responsible for its therapeutic activities. It has been reported that the main compounds in coriander seeds are essential oils [14], flavonoids [15], and carbohydrates [16]. However, there is limited literature on carbohydrate isolation from coriander seeds. Essentially, polysaccharides have attracted a worthy interest owing to their multiple physiological and biological properties, including antiproliferative, antioxidant, antimicrobial, hyperglycemic, wound-healing [17], and hepatoprotective effects to protect liver injuries induced by various chemicals [18].

Based on this background information, this current work aimed to assess, for the first time, the antioxidant capacity of water-soluble polysaccharides from coriander fruit and its hepatoprotective ability against cadmium-induced toxicity using a rat model. It was also interesting to characterize isolated carbohydrates via FTIR, DSC, NMR (1D and 2D), UV–vis, GC-MS, and macromolecular properties.

## 2. Materials and Methods

### 2.1. Plant Raw Material

A Tunisian variety of mature *Coriandrum sativum* seeds (Apiaceae family) was purchased from the Korba region in northeastern Tunisia in June 2020 (Lat. 36340 38.22″ N; Long. 10510 29.63″ E). Plant identification was carried out by a botanist. Afterward, seeds were milled to obtain a thin powder, defatted with petroleum ether (20%) for 4 h, and then left to dry at 40 °C for 48 h to obtain the defatted coriander seeds’ flour.

### 2.2. Chemicals

The different chemical reagents, including DPPH (2,2-diphenyl-1-picrylhydrazyl), Folin-Ciocalteu’s reagent, standard monosaccharides (xylose, glucose, rhamnose, arabinose, mannose, galactose, and fructose), trifluoroacetic acid (TFA), and thiobarbituric acid (TBA) were acquired from Sigma–Aldrich (St. Louis, MO, USA). Cd was obtained from CPI International (Santa Rosa, CA, USA). The various solvents used in this study were also Sigma–Aldrich products and they were used as received without further purification. 

### 2.3. Polysaccharides Extraction and Purification Procedure

Coriander seeds were depigmented using ethanol solvent for 2 days by a soxhlet to eliminate small molecules and then left to dry at 40 °C for 48 h. After that, polysaccharides were extracted using hot water: 20 g of seed flour was mechanically stirred in distilled water for 60 min at 80 °C and cooled. The obtained mixture was filtered and centrifuged (4000 rpm, 15 min). The obtained extract was precipitated with 3 volumes of 96% ethanol and left for 12 h at 4 °C. In order to remove proteins, the precipitates were melted and mixed with Sevag reagent. Then, dialysis against water was performed for the aqueous part at 4° C for 72 h via a dialysis membrane (cut-off 14 kDa). Lastly, dialysates were lyophilized to obtain the coriander polysaccharide (CPS).

### 2.4. Polysaccharides Characterization

#### 2.4.1. Colorimetric Assays

The polysaccharide amount of CPS was evaluated using the phenol-sulfuric acid method. The uronic acid amount was estimated using the carbazole method. The protein rate was calculated via the Lowry procedure, and the total amount of phenolics in carbohydrates was found using the Folin-Ciocalteu assay [19].

#### 2.4.2. DSC, UV–vis and FT-IR Experiments

The CPS infrared spectra were obtained by an Attenuated Total Reflectance (ATR-FTIR) spectrophotometer (PerkinElmer, Waltham, MA, USA) with a resolution of 4 cm^−1^ and scanning a wavelength from 400 to 4000 cm^−1^, and data were resolved using the Omnic 7.2 Software.

The CPS UV–vis analysis was carried out via a spectrophotometer (UV-2501PC; Shimadzu, Tokyo, Japan), and purified water was used as reference.

The DSC (differential scanning calorimetry) was performed using a PerkinElmer Pyris Diamond DSC differential scanning calorimeter (PerkinElmer; Waltham, MA, USA). Approximately 13 mg of the CPS was used for the analysis. The temperature range was 25–300 °C at a fixed heating rate of 10 °C/min.

#### 2.4.3. NMR Analysis

The structural analysis of CPS was realized by ^1^H NMR and ^13^C NMR, applying a BRUKER-ASX300 instrument (Bruker Inc., Rheinstetten, Germany). CPS was dissolved in D_2_O (99.96%). The (^1^H and ^13^C) NMR spectra were well-recorded, respectively, at 300 and 75 MHz frequencies. Two-dimensional experiment (COSY and HSQC) spectra were also carried out.

#### 2.4.4. Monosaccharide Composition

Polysaccharide fraction biochemical composition was determined according to an earlier procedure [20]. CPS sugar analysis was obtained after hydrolyzing with 1 mL of trifluoroacetic acid TFA, 2 M (at 70 °C, 2 h) and converting it to trimethylsilyl; then, a GC-MS analysis (Varian CP-3800 gas chromatograph; Varian, Inc., Santa Clara CA, USA) was performed. The analytical parameters were: injector (at 250 °C) and transfer line (at 240 °C); the temperature in the oven was increased from 60 to 240 °C with a rate of 3 °C/min; the helium gas rate was set at 1 mL/min.

#### 2.4.5. Size Exclusion Chromatography

CPS analysis was performed by SEC equipped with a Multi-Angle Light Scattering MALS (HELEOS II; Wyatt Technology, Santa Barbara, CA, USA), a differential refractive index DRI (RID 10A Shimadz, Tokyo, Japan), and a viscometer detector VD (Viscostar II, Wyatt Technology, Santa Barbara, CA, USA). The SEC apparatus includes a pump (LC10 Ai; Shimadzu, Tokyo, Japan) with lithium nitrate solution (LiNO_3_, 0.1 mol/L) as eluent. For this assay, CPS was dissolved in a 0.1 mol/L LiNO_3_ solution at a concentration of 2 g/L for 24 h stirring, well-filtered at 0.45 μm. Data were processed using the binding software of the Astra 6.1 package. Molecular weights were obtained with the Zimm order 1 method using angles from 34.8° to 142.8° and with a dn/dc of 0.15 mL/g. Analysis from VD used the Einstein/Simha formula for the hydrodynamic radius (R_h_) calculus [21].

### 2.5. Antioxidant Activity Assays

Two different methods were employed to evaluate CPS antioxidant potential. The capacities were performed in triplicate, and the average values were calculated.

#### 2.5.1. DPPH Test

CPS DPPH free radical scavenging ability was carried out [22] with few modifications. The absorbance of the different samples was well-measured at 517 nm with a UV–vis spectrophotometer (UV-2501PC; Shimadzu, Tokyo, Japan).

#### 2.5.2. FRAP Assay

The FRAP test was carried out [23]. Reaction mix absorbance was performed at 700 nm. A typical curve was plotted with various concentrations of FeSO_4_·7H_2_O (25–2000 μmol/L). Results were presented as antioxidant concentration with ferric-reducing power equivalent to FeSO_4_·7H_2_O (1 μmol/L).

### 2.6. In Vivo Hepatotoxicity and Cytoprotective Assays

#### 2.6.1. Animals and Experimental Design

Animals were accommodated corresponding to confidentiality Committee and the Ethics (EEC) 609/86 Directives adjusting the experimental animals’ welfare, and the experiments were assented by the local ethics committee of the Biotechnology Institute of Monastir University (Ref: CER-VS/ISBM 022/2020).

Male Wistar rats with an average weight of 150 g and an age of 6 weeks were obtained from our own breeding colony and were housed in cages at 22 ± 2 °C and a relative humidity of 55 ± 20%. A natural twelve-hour day/night cycle was implemented. Pellet diet food and water ad libitum were accessible.

Following a week of acclimatization, rats were arbitrarily sectioned into 4 experimental groups (three mice for each, *n* = 3) and treated using oral gavage feeding:

- Group. 1; (control group) receiving 1 mL of physiological saline (37 °C) daily;
- Group. 2; receiving 250 mg kg^−1^ b.w. dose of CPS daily;
- Group. 3; receiving 1 mL of 4 mg kg^−1^ b.w. dose of cadmium on the last day;
- Group. 4; receiving 250 mg kg^−1^ b.w. dose of CPS daily and 1 mL of 4 mg kg^−1^ b.w. dose of cadmium on the last day.

In our work, Cd was dosed orally to rats using gavage as it is the highest form of exposition to cadmium in animals/humans. All investigated groups were dosed (once a day) for 15 successive days. Following 24 h of fasting guided by the previous administration, rats were anesthetized and sacrificed for tissue collection. The different samples’ dose decision was based on previous study with slight modifications [2]. Livers were dissected and instantly frozen in liquid nitrogen and subsequently saved at −80 °C until analysis. Tissues were integrated in ice cold Tris-HCl buffer (100 mM, pH = 7.5) and then centrifuged (5000× *g*, 15 min) at 4 °C.

#### 2.6.2. Determination of Catalase CAT Assay

CAT trial in liver supernatant was performed using the Clairbone method [24]. Briefly, samples were analyzed at 240 nm absorbance to control H_2_O_2_ concentration. The mixture of samples included PBS solution (100 mM, pH = 7), 200 mL H_2_O_2_ (0.5 M), and 20 μL of treated sample. 

#### 2.6.3. SOD Assay

SOD assay was measured at 325 nm in a liver supernatant by measuring the auto-oxidation inhibitory effect of pyrogallol [25]. The one unit of enzyme effect (U) was defined as enzyme amount that exhibited 50% inhibition of the auto-oxidation rate of pyrogallol in 1 mL solution and the SOD assay was expressed in U mg^−1^ protein.

#### 2.6.4. Lipid Peroxidation (MDA)

The complex TBA-MDA was quantified by HPLC combined with fluorescence detection to determine the CPS in liver homogenate tissue. The analysis of the TBA-MDA adduct was performed on an Ultrasep-C18 column. To adjust the pH of the methanol/water (40:60 *v*/*v*) solvent to 8.3, a KOH solution (0.5 M) was added to this mobile phase. CPS amounts were well-expressed as μmol malondialdehyde contents MDA/mg protein.

#### 2.6.5. Total Protein Content

The total protein concentration in liver supernatants was estimated according to the Bradford method [26].

### 2.7. Statistical Analysis

All trials were evaluated in three independent experiments (*n* = 3) and were expressed as mean ± standard deviation (SD). Treated animals were compared with the group control (non-treated) using the one-way ANOVA. Data statistical variations were obtained through the Student’s *t*-test (software driver 16.0). Differences in results were deemed significant at *p* ≤ 0.05.

## 3. Results and Discussion

### 3.1. CPS Extraction and Identification

Conventional water extraction is the most common method to extract polysaccharides from plants. Usually, the efficacy of carbohydrate extraction from plant sources relies on many factors (liquid–solid ratio, time and temperature of extraction). In our research, we selected hot water extraction to extract CPS polysaccharides. The yield of polysaccharide powder obtained from coriander seeds was 9.10%. The same result was obtained for the aniseed polysaccharides [27]. Table 1 shows the results of the chemical analysis of CPS polysaccharide. CPS sugar composition and uronic acid contents were 62.2% and 9.8%, respectively. However, no protein or polyphenols were found.

Gas chromatography coupled with mass spectrometry (GC-MS) of CPS was carefully performed. According to previous works, some polysaccharides isolated from seeds are mostly composed of arabinans [28], glucans, xylans, and galactans [29]. In our study, results showed that CPS polysaccharide was composed of different monosaccharides, namely rhamnose, arabinose, xylose, fructose, mannose, glucose, and galactose (3.6%, 6.2%, 8.8%, 5.2%, 17.7%, 25.6%, and 32.9% of relative mass, respectively) (Table 1). These results were also found in some *Apiacea* plants (presented in seeds), particularly in *Pimpinela anisum* and *Anethum graveolens* seeds [27,30]. It is probable that, due to the richness in monosaccharides (galactose and glucose), CPS is mainly a polysaccharide of the glucogalactan type.

**Figure 1:**
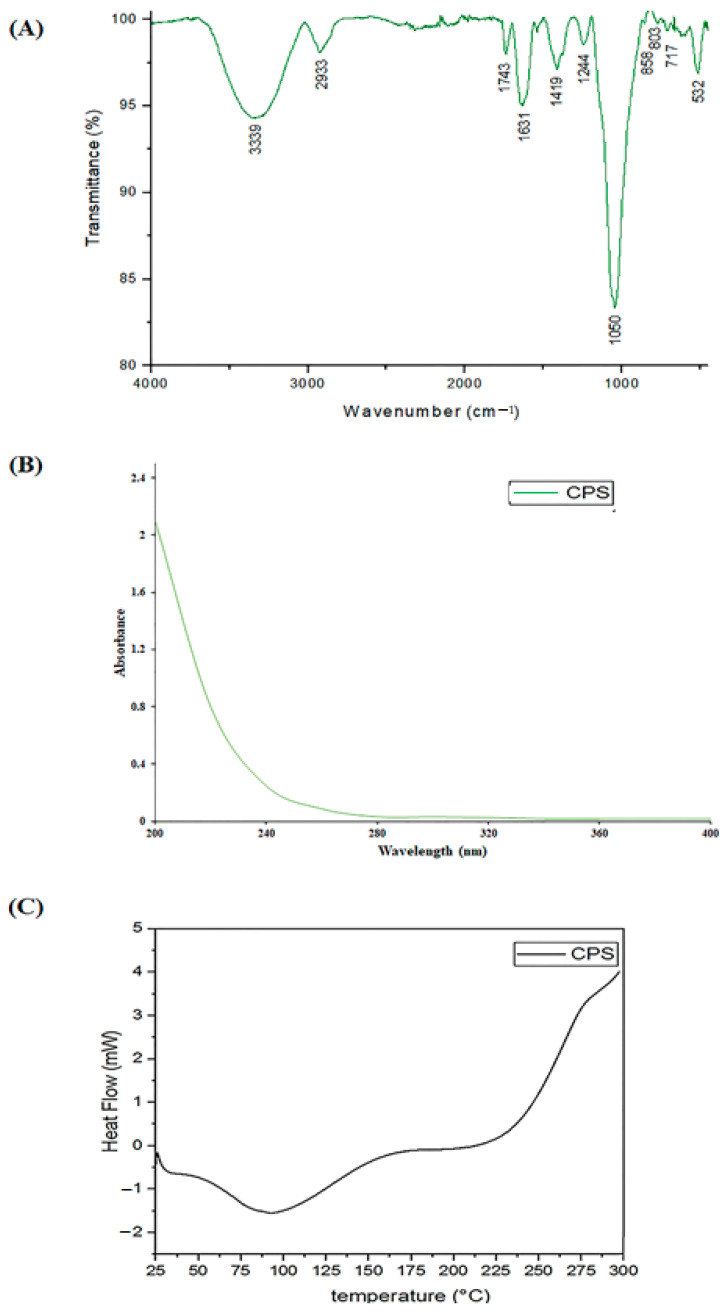
(**A**): FTIR spectrum of CPS; (**B**): UV–vis spectrum of CPS; (**C**): DSC thermogram of CPS.

The FTIR spectrum of CPS (Figure 1A) showed typical polysaccharide absorption bands. In effect, a wide absorption band at 3339 cm^−1^ was assigned to the hydroxyl groups stretching (O-H). An absorption band in 1743 cm^−1^ was attributed to the C=O stretching vibration of O-acetyl groups. The band at 1631 cm^−1^ was specified in carboxylate and carboxylic groups’ stretching band (COO-). A detected band centered at 1419 cm^−1^ belayed the attendance of uronic acid units. In addition, the spectrum reveals the presence of specific sugar bands (spectral fingerprints of sugars) in the region between 1000 and 1200 cm^−1^. Indeed, a strong band near 1035 cm^−1^ showed the C-O-C stretching vibration of the glycosidic structure [31]. Absorption bands in the range of 1000–1100 cm^−1^ indicated the presence of pyranose. Absorption bands at 858 cm^−1^ and 803 cm^−1^ submitted that both α- and β-configurations jointly exist in CPS [32].

The CPS UV spectrum is represented in Figure 1B. No peak in the 260–280 nm range was detected, showing that CPS have no protein or nucleic acid. This finding is consistent with the chemical analysis described below (Table 1).

Thermal analysis was achieved to appreciate the thermal behavior and changes in the physical and chemical properties of CPS polysaccharide. Figure 1C represents the DSC thermogram of CPS. In fact, the curve for the temperature range 25–300 °C showed that the thermal behavior of CPS represents two distinct events. The first endothermic transition showed a broad melting transition spanning from 60 to 125 °C, which is associated with the loss and evaporation of free water. The second exothermic transition was observed at a temperature of about 240 °C. This transition is associated with the thermal decomposition and branching of the extracted polysaccharide [33]. CPS exhibited thermal stability indicating that they can withstand high-temperature processes related to the fields of functional foods and medicine.

**Figure 2:**
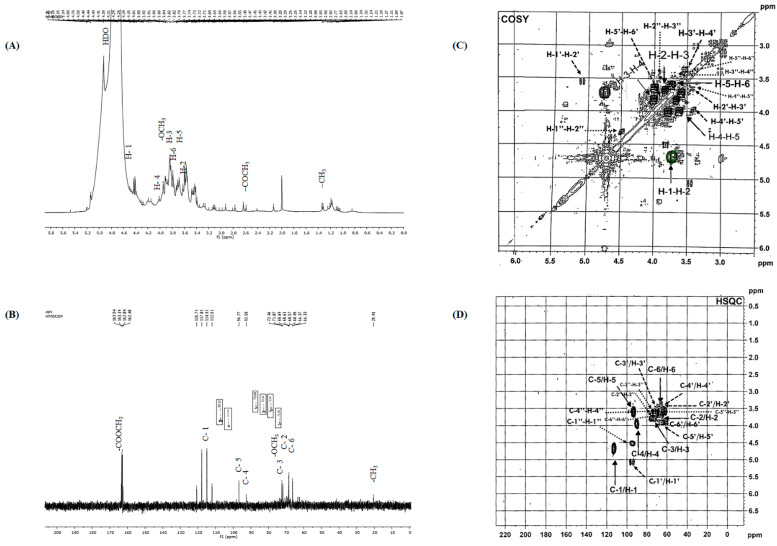
NMR spectra of CPS registered in D_2_O at 50 °C. (**A**): ^1^H NMR (300 MHz), (**B**): ^13^C NMR (75 MHz), (**C**): COSY, (**D**): HSQC.

NMR spectroscopy was non-destructive and an effective method to obtain structural information of polysaccharides [34]. Figure 2 shows the different 1D and 2D spectra of the CPS obtained by NMR analysis. All signals were assigned as completely as possible, based on GC-MS analysis and chemical shifts as described in literature. 

In fact, ^1^H NMR signals were assigned as shown in Figure 2A. In the anomeric region, the displacement of anomeric H-1 at δ5.20, 5.21, 5.15, 5.14, and 4.93 ppm was higher or lower than 5.0 ppm, indicating that both α- and β-configurations exist [32]. These data further ensured the infrared spectrum results. According to the relevant literature data [35], the anomeric proton signals δ4.70, 5.20, and 5.21 ppm are assigned to the proton H-1 of Gal*p*, Rha*p*, and Ara*f*, respectively. A large signal at 3.82 ppm was derived from methyl groups binding to carboxyl groups. Thus, the resonances at δ 1.34 ppm correspond to the methyl group of Rha*p* residue, which agreed with the monosaccharide analysis [34]. The signals at about 2.59 ppm are assigned to the methyl protons of the acetyl groups. The strong signal around 4.93 ppm is attributed to the HDO solvent. [36]. Moreover, six chief signals were detected and assigned as Gal*p* residues: H1, 4.70 ppm; H2, 3.69 ppm; H3, 3.79 ppm; H4, 4.01 ppm; H5, 3.73 ppm; and H6, 3.74 ppm, respectively [37,38]. The rest of signals might correspond to the absorption of Glc*p* residues (H1′: 5.14; H2′: 3.55; H3′: 3.56; H4′: 3.41; H5′: 3.92; H6′:3.84) [39]. Thus, ^1^H NMR spectra show signals at δ 4.50 (H1″), 3.82 (H2″), 3.57 (H3″), 3.58 (H4″), 3.60 (H5″), and 3.43 (H6″), attributed to Man*p* residues [40].

The ^13^C NMR spectrum of CPS exhibited significant complexity (Figure 2B), showing two kinds of anomeric configurations for monosaccharide residue. In fact, signals in the range of 55 to 86 ppm can be assigned to sugars C2–C6, referring to the literature data [41]. The signals derived from anomeric carbons generally appear in the 90–115 ppm region [42]. There were five anomeric carbon signals at δ117.81, 114.91, 112.01, 96.77, and 92.77 ppm, and these were attributed to the C1 of five anomeric residues. The ^13^C chemical shift at δ 163.54 ppm was assigned to carboxyl carbons [34]. Moreover, the chemical shift at δ 20.41 ppm was due to the methyl groups [43]. Thus, the signal at 114.91 ppm was attributed to the C1 of Gal*p*; signals of Gal*p* C2, C3, C4, C5, and C-6 were at 71.87, 72.46, 92.58, 96.77, and 66.33 ppm, respectively [44]. Additionally, the intense signal around 72.46 ppm corresponds to the CH_2_-O and CH-O groups. The signal at 97.85 ppm was assigned to the C1′ of Glc*p*; the signals of Glc*p* C2′, C3′, C4′, C5′, and C6′ were at 68.69, 68.61, 68.57, 68.49, and 66.37 ppm, respectively [45]. Thus, the signals of Man*p* C1″, C2″, C3″, C4″, C5″, and C6″ were at 98.12, 71.56, 72.80, 73.42, 73.68, and 63.23 ppm, respectively [40]. This finding was in accordance with those obtained through monosaccharide composition analysis.

The 2D COSY spectrum of CPS (Figure 2C) was achieved to afford diverse cross points among anomeric protons H1 and H2 at 4.70/3.69 ppm, H2 and H3 at 3.69/3.79 ppm, H3 and H4 at 3.79/4.01 ppm, H4 and H5 at 4.01/3.73 ppm, and H4 and H5 at 3.73/3.74, corresponding to Gal*p* residues [37]. Moreover, the cross-peaks were found δ5.14/3.55, 3.55/3.56, 3.56/3.41, 3.41/3.92, and 3.92/3.84 in the case of Glc*p* residues. Thus, main residual cross-peaks were observed δ4.50/3.82, 3.82/3.57, 3.57/3.58, 3.58/3.60, and 3.60/3.43 for the Man*p* residues. These outcomes are consistent with reports from several studies [27,34].

To obtain additional accurate structure information on CPS, heteronuclear singular quantum correlation (HSQC) was carried out. These spectroscopic correlations allowed us to attribute the chemical shifts of the protons and corresponding carbons (Figure 2D). The correlations at δ114.91/4.70, 71.87/3.69, 72.46/3.79, 92.58/4.01, 96.77/3.73, and 66.33/3.74 ppm corresponded to the C1/H1, C2/H2, C3/H3, C4/H4, C5/H5, and C6/H6 of Gal*p* residues, respectively. Additionally, several cross-peaks were observed, including at δ97.85/5.14, 68.69/3.55, 68.61/3.56, 68.57/3.41, 68.49/3.92, and 66.37/3.84 ppm, which correspond to the C1′/H1′, C2′/H2′, C3′/H3′, C4′/H4′, C5′/H5′, and C6′/H6′ of Glc*p* residues, respectively. Additionally, cross-peaks were detected, including at δ98.12/4.50, 71.56/3.82, 72.80/3.57, 73.42/3.58, 73.68/3.60, and 63.23/3.43 ppm, which correspond to the C1″/H1″, C2″/H2″, C3″/H3″, C4″/H4″, C5″/H5″, and C6″/H6″ of Man*p* residues, respectively.

**Figure 3:**
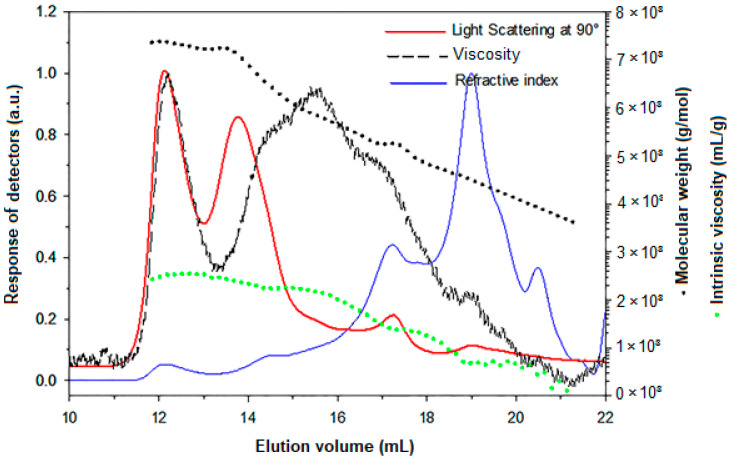
Elution profiles by SEC with refractive index (full blue line), viscosity (black dash line), and LS at 90° (full red line) of CPS with molecular weight (black circle) and intrinsic viscosity (green dash line) distributions in LiNO_3_ 0.1 mol/L.

The physicochemical parameters of CPS were determined through SEC/MALS/DRI/VD. Figure 3 displays the curves of molecular weight and intrinsic viscosity distributions with light scattering at 90°, DRI, and specific viscosity profiles for CPS in LiNO_3_ 0.1 mol/L.

The SEC chromatogram can be divided into two populations. Before 15 mL, the light scattering response was significant, with a low concentration signal. For this population (7% of the total mass), the chains had a molecular weight higher than 500,000 g/mol with hydrodynamic radii between 10 and 100 nm. Chains eluted after 15 mL were the principal population (93% of the total mass) with low molecular weights and sizes. Consequently, the calculation of the number and weight average molecular weights on the whole sample shows a large dispersity (Mn = 16 kDa; Mw = 1000 kDa; Đ = 62). With the viscometer detector, we obtained the weight average intrinsic viscosity ([η] = 34 mL/g) and the hydrodynamic weight radius (R_h_ = 10 nm). The length of most CPS chains is relatively short, which is generally favorable for obtaining interesting biological properties.

The molecular weight dependences of intrinsic viscosity (via Mark Houwink Sakurada) and hydrodynamic radius are well known to provide information on the chain conformation. The obtained data for CPS ([η] = 0.037 M^0.58^ and Rh = f(M^0.49^)) showed a random coil conformation.

### 3.2. Antioxidant Capacities

**Figure 4:**
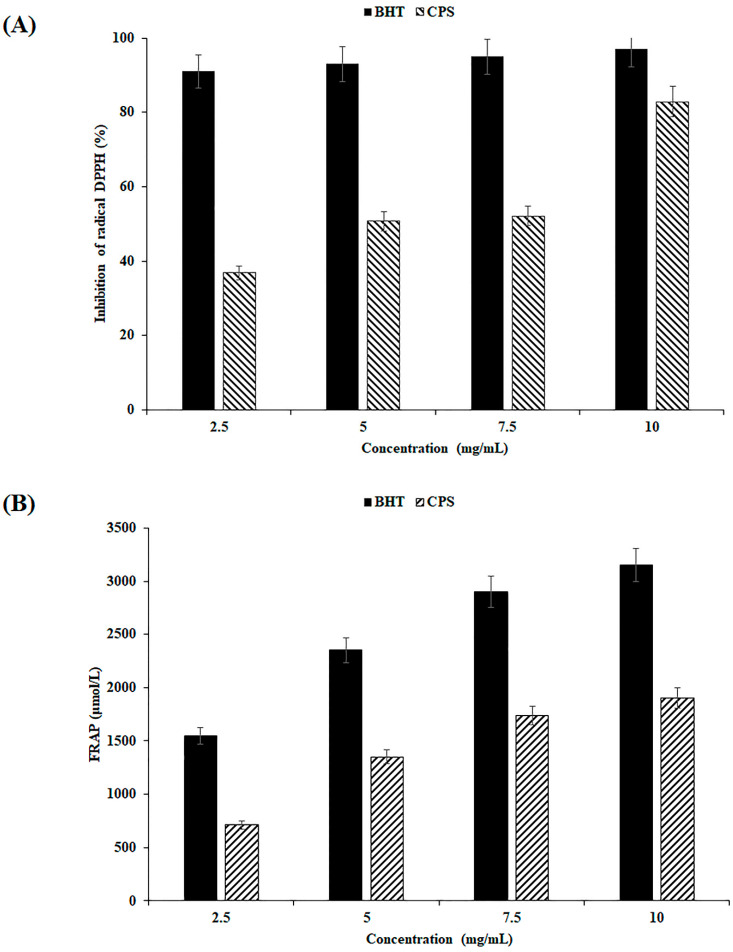
Estimation of antioxidant activities of CPS: (**A**) Scavenging ability on DPPH radicals; (**B**) Reducing power. Butylated hydroxytoluene (BHT) is employed as a reference. Each value is expressed as mean ± SD (*n* = 3).

The DPPH test is the most commonly used method to estimate the antioxidant effect of extracts. DPPH is a stable free radical whose absorbance decreases when antioxidants donate hydrogen or proton radicals, and it neutralizes itself. In our research, CPS may work as an antioxidant because it can convert the purple DPPH radical compound into a colorless product. In effect, Figure 4A reveals that the reference standard possessed an obvious inhibitory ability. In addition, at a concentration of 2.5 to 10 mg mL^−1^, CPS exhibited a scavenging activity on DPPH free radicals. At 10 mg/mL, CPS showed the broadest DPPH scavenging potential, with 82.89% of inhibition. The free radical scavenging inhibitory effect on CPS can be illustrated via the carboxyl and hydroxyl groups attached to monosaccharide units that can contribute protons to reduce DPPH free radicals.

The FRAP assay is broadly used to evaluate the antioxidant-reducing ability. This test is based on a ferricyanide (Fe^3+^) complex reduction to ferrous (Fe^2+^) complex with the highest absorbance (700 nm). This property is due to the presence of reducing agents that can easily donate electrons. A maximal reaction mixture absorbance designated an important reducing power of the antioxidant extracts. The (Fe^2+^) complex concentration in the extracts is the main indicator of its potential reduction activity. Figure 4B illustrates that for CPS and the reference (BHT), reducing power elevated clearly with the increase in CPS concentrations. Results show that CPS exhibits significant reducing activity at 10 mg/mL with 1900 μmol/L compared with the BHT reference.

### 3.3. Effects of Coriandrum sativum Seed on CdCl_2_-Induced Hepatotoxicity

SOD and CAT are widely used as biomarkers of the pro-oxidant potential of xenobiotics. These proteins act synergistically to reduce the production of reactive oxygen free radicals (ROS) and prevent damage caused by lipid peroxidation and intermediate metabolic products from causing harm [46].

Usually, the liver and erythrocytes contain sufficient scavengers such as SOD, CAT, GSH, GPx, etc., which can prevent free radical damage. SOD plays a key role in protecting cells from oxidative damage by converting superoxide free radicals into hydrogen peroxide, which is then metabolized by CAT to break down hydrogen peroxide into molecular oxygen and water. Thus, combined activities of SOD and CAT could possibly be essential to reduce oxidative stress [47]. Malondialdehyde (MDA) is one of the principal products of peroxidized polyunsaturated fatty acids oxidation. MDA can create injurious stress in cells and form homopolar protein adducts, also noted as advanced lipoxidation end products, which are generally seen as markers for estimating oxidant stress levels in organisms [48]. In this work, MDA, CAT, and SOD were chosen to determine in vivo antioxidant effects.

#### 3.3.1. Lipid Peroxidation (MDA)

**Figure 5:**
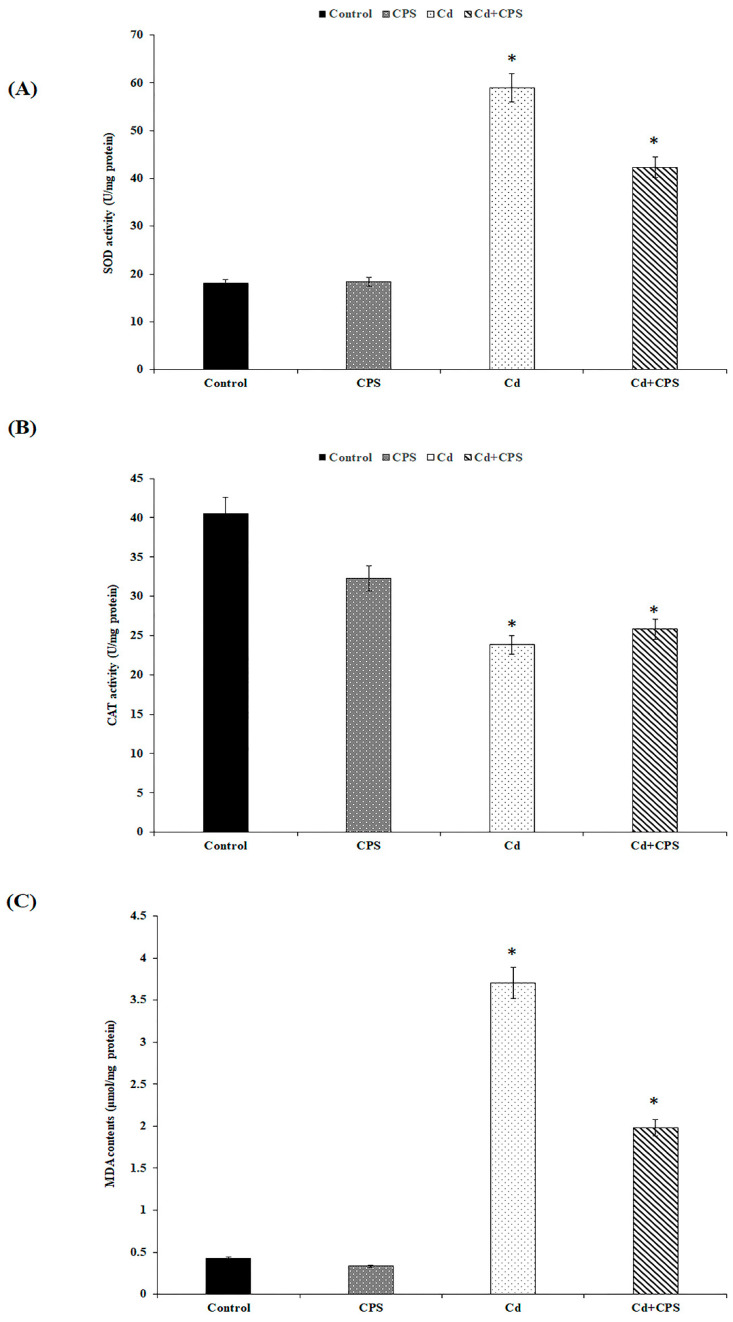
Mean (±SD) impact of CPS on Cd-induced damage in enzyme antioxidant concentration in liver tissues of control and treated rats (*n* = 3): (**A**): SOD enzyme, (**B**): CAT enzyme, and (**C**) MDA content in liver tissues of control and treated rats. * values are significantly different from the control at *p* ≤ 0.05.

As shown in Figure 5A, SOD activity was highly stimulated when animals were exposed to Cd, which suggests that the metal exposure enhances oxygen free radical production and stimulates an antioxidant response to scavenge free radicals.

Our data showed that the combination of CPS with Cd exhibits an interesting reduction in antioxidant activity. This, therefore, suggests that the polysaccharide extract is an effective ROS scavenger, which counteracts the peroxidative potential of the xenobiotic.

#### 3.3.2. CAT Activity

Enzymatic antioxidants including CAT and SOD maintain a balanced redox state to form the initial line of defense against ROS in the body and act as a biomarker for their production. In this way, CAT accelerates the decomposition of H_2_O_2_ to generate non-toxic oxygen and water, and SOD can transform superoxide anions into hydrogen peroxide [49].

Regarding CAT activity (Figure 5B), we show that the enzyme activity in the hepatic tissue of Cd-treated animals decreased compared to the control group and the CPS-treated group. This result could be assigned to the non-activation of the enzyme or the variation of the assembly of enzyme subunits under Cd exposure. The combination of Cd with the polysaccharide extract relatively improves enzyme activity, suggesting that this compound enhances antioxidant response by scavenging free radicals and protects hepatic tissue against Cd toxicity.

#### 3.3.3. MDA Production

The level of malondialdehyde is deemed a reliable indicator of membrane lipid peroxidation. In fact, this compound is a system-induced degradation product during lipid peroxidation, marking the free radical attack of polyunsaturated lipids. Thus, the rise in the MDA content is a notable indicator of cell membrane lipid peroxidation. Therefore, inhibition of lipid peroxidation is deemed to be the most valuable indicator of antioxidant capacity. The results indicated that hepatic MDA levels increased after cadmium exposure compared to the healthy group and reached 3.70 μmol MDA/mg protein in the Cd-treated liver (group 3) versus 0.33 μmol in the untreated liver (group 1) (Figure 5C). This increase validates the production of notable lipoperoxidation. The combination of CPS and Cd (group 4) has significantly lowered the creation of MDA when contrasted with Cd-treated animals. This reduction shows that CPS exerts a preventive and antioxidant effect against lipoperoxidation, which points to the ROS scavenging role performed by CPS polysaccharides.

## 4. Conclusions

**Figure 6:**
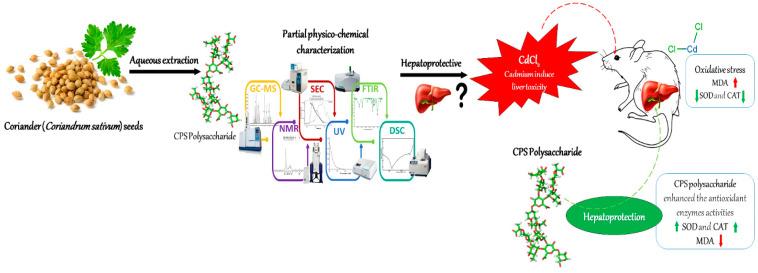
Graphical abstract and schematic layout of this study.

In this study, the polysaccharide was effectively isolated, by hot water extraction, from *Coiandrum sativum* seeds with a yield of 9.1%. Further, characterization via UV–vis, FTIR, DSC, GC-MS, and NMR showed the presence of several monosaccharides such as Gal, Glc, and Man, which are the main CPS sugars. Moreover, CPS revealed a remarkable radical scavenging effect on DPPH and FRAP free radicals. The results revealed the ability of CPS as an efficient agent in resisting oxidative stress and minimizing Cd-induced oxidative stress in animal liver tissues (Figure 6). In addition, the obtained outcomes indicate that CPS allows protection against Cd-induced hepatotoxicity through ROS scavenging and antioxidant activities. Therefore, CPS may be an effective new source of natural antioxidants with potent value for therapeutic agents and healthy foods.

## Figures and Tables

**Table 1:** CPS monosaccharide compositions results.

	Neutral Sugar (%)	Uronic Acid (%)	Protein (%)	Monosaccharide Composition (%) ^a^						
				Ara	Rha	Xyl	Man	Fru	Gal	Glc
**CPS**	62.2	9.8	-	6.2	3.6	8.8	17.7	5.2	32.9	25.6

Ara = arabinose, Rha = rhamnose, Xyl = xylose, Man = mannose, Fru = fructose, Gal = galactose, Glc = glucose. ^a^ Molar percentage (%) determined by GC-MS.

## Data Availability

Data is contained within the article.
